# Associations between the dietary inflammatory index, body mass index, and waist-to-height ratio and diagnosed and undiagnosed diabetes mellitus in adults in Guangxi, China

**DOI:** 10.1186/s12889-025-25942-9

**Published:** 2025-12-26

**Authors:** Zhongyou Li, Yan Li, Hai Li, Zhifeng Fang, Yuzhu Chen, Xiaopeng Li, Qiulan Qing, Weiwen Zhou, Jiongli Huang

**Affiliations:** 1 Department of Preventive Medicine, School of Public Health and Management, Guangxi University of Chinese Medicine, Nanning, 530200 China; 2 Guangxi Zhuang Autonomous Region Center for Disease Control and Prevention, Nanning, 530028 China

**Keywords:** Dietary inflammation index, Diagnosed diabetes mellitus, Undiagnosed diabetes mellitus, Body mass index, Waist-to-height ratio

## Abstract

**Background:**

The relationships between the dietary inflammatory index (DII), body mass index (BMI), and waist‒to-hip ratio (WtHR) and both diagnosed diabetes mellitus (DDM) and undiagnosed diabetes mellitus (UDDM), as well as the contributions of these factors, have not yet been comprehensively evaluated. This study aimed to investigate this association and elucidate the roles of DII, BMI, and the WtHR in the development of diabetes.

**Methods:**

This was a cross-sectional study involving 3687 participants aged 18 to 69 years were selected from the China National Nutrition and Health Survey (CNNHS 2010 to 2013) and the China Adult Chronic Disease and Nutrition Surveillance (CACDNS 2015) in Guangxi. DII scores were calculated from a 3-day, 24-hour (3d 24 h) dietary survey combined with the weighing method. Unconditional logistic regression, restricted cubic spline (RCS), and weighted quantile sum (WQS) models were utilized to assess the associations between DII, BMI, WtHR, and both DDM and UDDM.

**Results:**

The overall prevalence of TDM was 6.6%, comprising 40.6% DDM and 59.4% UDDM cases. Compared with Q1 (the lowest proinflammatory group), subjects in Q4 and Q5 exhibited significantly higher TDM risk, with ORs (95% CI) of 1.65 (1.06, 2.56) and 1.88 (1.21, 2.92), respectively. This association was particularly pronounced in UDDM, where a significant dose-response relationship emerged (P-trend = 0.006), with Q5 demonstrating the highest diabetes risk which OR (95% CI) was 1.98 (1.16, 3.40). DII scores and the risks of TDM and UDDM were linear (all *P* values < 0.05) but no association between the DII and DDM risk. There is a nonlinear relationship between BMI and DDM risk, a linear relationship with UDDM risk, and significant positive correlations between the WtHR and both DDM and UDDM risk. WtHR emerged as the primary contributor (weight = 0.69) in participants with DDM. For participants with UDDM, DII emerged as the primary determinant, outweighing BMI (weight = 0.37 vs. 0.35) and the WtHR (weight = 0.28).

**Conclusions:**

A high-proinflammatory diet is significantly linked to increased risks of both DDM and UDDM. BMI and the WtHR also exert substantial yet differential influences.

**Supplementary Information:**

The online version contains supplementary material available at 10.1186/s12889-025-25942-9.

## Introduction

From 1990 to 2022, the number of adult diabetes cases worldwide increased from 200 million to 828 million [1]. This alarming trend is driven primarily by the increasing prevalence of type 2 diabetes, which accounted for 90% of all diabetes mellitus (DM) cases in 2021 [2]. Type 2 diabetes represents a significant and growing global health burden, with its prevalence rapidly increasing worldwide [3, 4]. In 2022, China had 148 million adult diabetic patients, 18% of the global total, ranking second [1]. With approximately 140 million type 2 diabetes patients, China leads globally, posing a significant public health challenge [5]. The 11th edition of the “IDF Global Diabetes Map” indicates that 42.8% of diabetes patients worldwide remain undiagnosed, and there are 251 million diabetes patients who are undiagnosed [6]. In China, this proportion is even higher (49.7%), estimated at 73.5 million UDDM cases in China, which is among the highest reported cases globally [6, 7]. Moreover, the prevalence of type 2 diabetes in China is increasing, exacerbating this challenge [8, 9]. Individuals with UDDM face an increased risk of complications such as cardiovascular disease, nephropathy, retinopathy, risk of death due to the lack of timely medical intervention [7, 10].

Among the modifiable risk factors contributing to the development of type 2 diabetes, diet and obesity have emerged as critical determinants [11]. Addressing these risk factors is imperative for mitigating the burgeoning diabetes epidemic and its associated health burdens [4, 12–15]. The Dietary Inflammatory Index (DII) evaluates the inflammatory potential of an individual’s diet. Pro-inflammatory diets, reflected by a high DII, can promote chronic low-grade inflammation by disrupting the intestinal barrier and altering plasma metabolites. This inflammatory state subsequently reduces insulin sensitivity and elevates the risk of chronic conditions, including type 2 diabetes mellitus. Furthermore, such diets elevate circulating levels of inflammatory factors, including interleukin-6 (IL-6) and tumor necrosis factor-α (TNF-α). These cytokines inhibit insulin signal transduction, impair pancreatic β-cell function, and thereby accelerate the progression from abnormal blood glucose to overt diabetes [16, 17]. Against this backdrop, the DII has gained attention as a valuable tool for assessing an individual’s overall dietary inflammatory [18–21]. High levels of DII have been shown to increase the risk of developing diabetes [16] and promote the development of obesity [22], which further exacerbates insulin resistance and subsequently leads to an increased incidence of diabetes [23].Most previous studies [16, 24–30] support positive correlations of the DII with the TDM, DDM and gestational diabetes. However, two studies [31, 32] reported nonsignificant associations between DII and DM risk. These discordant findings may partially stem from the variations in the study population. What is more worth exploring is whether there exists a non-linear relationship between DII and DDM. The association between DII and UDDM are limited globally. Compared with DDM, those with UDDM had higher DII scores in the US general population [26]. Another study [16] reported that a higher DII increased the risk of UDDM. Patients with DM who have undergone hospital-based diagnosis and treatment have typically received dietary and behavioral health guidance, potentially altering their habits. In contrast, among individuals with UDDM, who remain unaware of their glycemic status and thus maintain original dietary patterns, the DII may exert a different influence. However, these studies did not establish a dose-response relationship between DII and UDDM and determine whether DII contributes differently to DDM and UDDM.

Obesity is another significant contributor to the development of DM [28, 33]. Higher BMIs and WtHRs are significant risk factors for diabetes [33, 34]. BMI is widely used but has limitations in accurately reflecting fat distribution, particularly abdominal fat accumulation, which is a significant risk factor for metabolic diseases such as diabetes [35]. It was proposed that obesity diagnosis should incorporate additional metrics, such as waist circumference, the WtHR, and the visceral fat area [36]. In recent years, the WtHR has emerged as a novel obesity assessment metric, gaining attention because of its ability to comprehensively reflect abdominal obesity by considering the ratio of waist circumference to height without being restricted by age or sex [33, 35, 37]. Several studies [38, 39] have demonstrated an association between the WtHR and the risk of diabetes and prediabetes. Consequently, in the early prevention of diabetes, the WtHR may offer significant advantages over BMI [26]. In fact, while both the Guidelines for the Diagnosis and Treatment of Obesity (2024) [40] and the Chinese Guidelines for the Prevention and Treatment of Diabetes Mellitus (2024) [41] utilize BMI as the primary criterion for obesity diagnosis, they also employ waist circumference (WC) to assess central obesity in adults (defined as WC ≥ 90 cm for males and ≥ 85 cm for females). However, it is recognized that relying solely on WC may not be the most appropriate method for evaluating body composition in populations with relatively shorter stature. Several studies in China [42, 43] have demonstrated the superior predictive and evaluative utility of WtHR in assessing diabetes risk. Nevertheless, research in this area remains limited in China.

Although studies have examined associations of DII, BMI, and WtHR with diagnosed or undiagnosed diabetes, the relative contributions of these interrelated factors and their potential nonlinear relationships remain incompletely characterized in Chinese populations. Therefore, this study used the data of CNNHS 2010 to 2013 and CACDNS 2015 to analyze the association between DII, BMI and the WtHR and between the DDM and UDDM in Guangxi, China. The specific aims of this study are as follows: (a) To investigate the linear and non-linear associations of DII, BMI, and WtHR with the risk of DDM and UDDM, respectively; (b) To focus on exploring the association of DII with the risk of DDM and UDDM; (c) To quantify the respective contribution of DII, BMI, and WtHR to the risk of DDM and UDDM.

## Methods

### Study area and study population

This study employed a cross-sectional survey design, utilizing data sourced from CNNHS 2010 to 2013 and CACDNS 2015 conducted in Guangxi. The surveys utilized a multistage stratified cluster random sampling approach. Specifically, five monitoring sites were selected from the CNNHS 2010 to 2013, and ten monitoring sites were chosen from the CACDNS 2015 in Guangxi.

First, both the CNNHS 2010 to 2013 and CACDNS 2015 were organized and implemented by the Institute of Nutrition and Health at the Chinese Center for Disease Control and Prevention, which also contributed to the study design. The dietary questionnaires and survey methodologies were consistent between the two surveys, and the same brands and models of equipment were used for all anthropometric measurements, including height, weight, waist circumference, and blood pressure. Second, the analysis of fasting blood glucose and blood lipids for both projects was conducted by qualified laboratories at county-level Centers for Disease Control and Prevention. All kits and technical protocols were supplied by the national project team, and laboratory personnel were required to pass a quality assessment using blind samples provided by the national quality control group before performing any tests. Third, in Guangxi, the same project team from the Guangxi Zhuang Autonomous Region Center for Disease Control and Prevention was responsible for the organization, implementation, and quality control of field operations in both surveys, ensuring continuity and consistency in staff training, technical standards, fieldwork, and quality assurance. We therefore combined and analyzed the samples from both surveys to increase sample size and enhance statistical power.

**Fig. 1:**
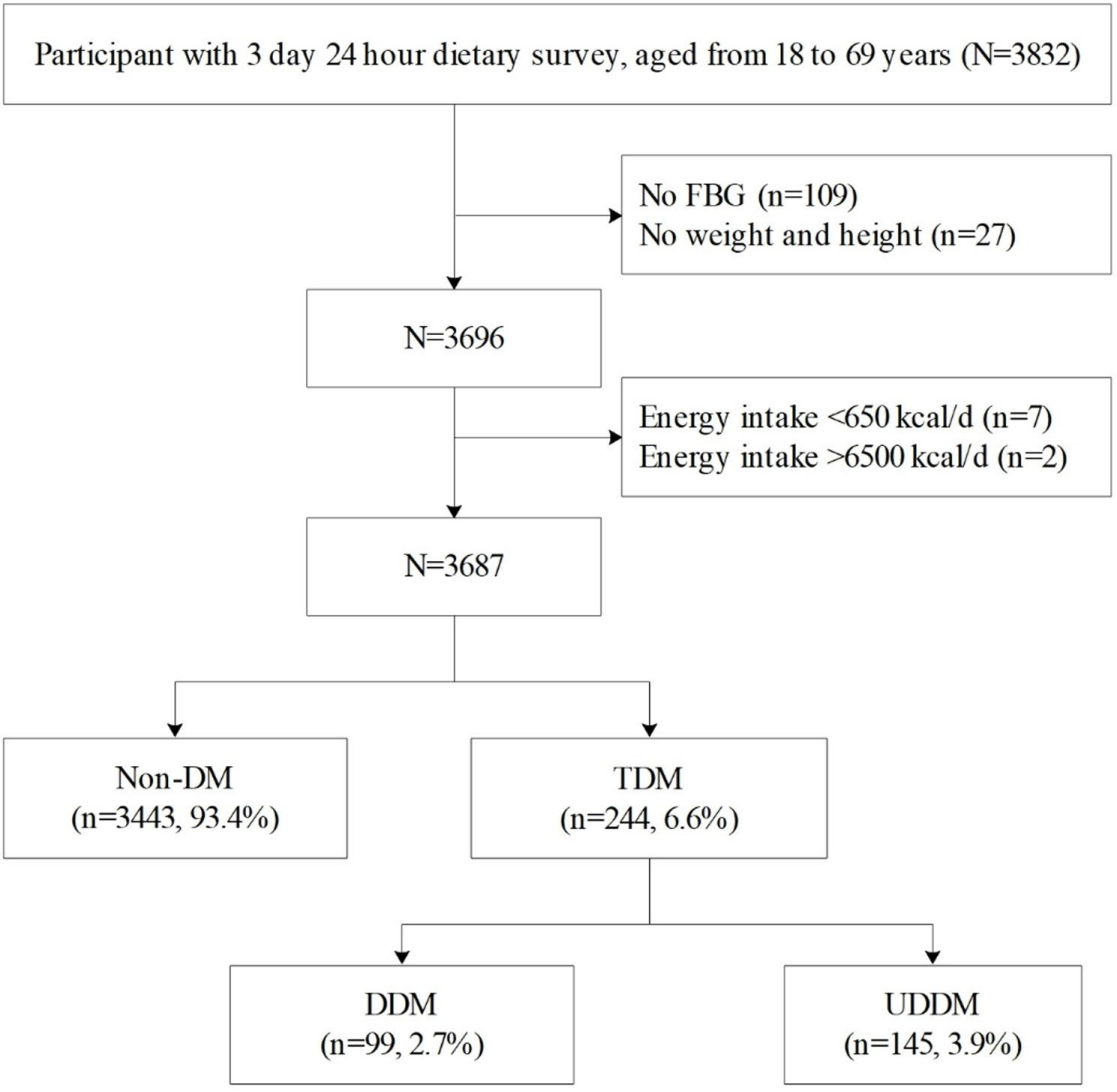
Flowchart of sample selection in this study

The family unit served as the fundamental unit of our sampling strategy. A comprehensive assessment battery was administered, encompassing a personal questionnaire, physical examination, blood biochemistry tests, and a dietary survey. The dietary survey employed both a 3-day 24-hour recall method and a weighing method to provide a detailed account of participants’ nutritional intake. Eligibility for participation was determined on the basis of the following criteria: (a) local permanent residents (for more than six months each year); (b) age between 18 and 69 years and (c) completion of the dietary survey, physical measurements, and blood tests. Subjects were excluded as follows: (a) pregnant women and Breastfeeding women; (b) those who suffer from mental disorders or are currently taking medications for mental disorders; (c) those who had incomplete questionnaires; (d) energy intake outside the range of 650 to 6500 kcal/day. Ultimately, the study included 3687 participants. Figure 1 presents a flowchart illustrating the sample selection process. This study was approved by the Guangxi Ethics Review Committee (GXIRB 20140003). All the subjects were fully informed and signed informed consent before the investigation.

### Dietary investigation

Qualified interviewers conducted in-home assessments of subjects’ dietary intake over the preceding 24-hour period, performing daily interviews for three consecutive days (2 weekdays, 1 weekend) via the dietary recall method. Throughout this period, the interviewers meticulously weighed the cooking oil and condiments consumed by each family member. The subjects’ proportional consumption of oil and condiments was determined by calculating their individual energy intake as a percentage of the total family energy intake. The caloric and nutritional contents of the reported food intake were quantified via the China Food Composition Table [44]. The methods of dietary survey and data calculation were described in our previous study [45].

### DII calculation

 The DII is a useful tool created by Cavicchia et al. [18] in 2009 and Shivappa et al. [19] in 2014 and was developed for use in different populations. The consumption of different foods (nutrients) is closely related to inflammation in the human body, with some nutrients capable of promoting an increase in inflammation, whereas others are associated with reducing the level of inflammation in the body [20].

The DII calculation has been described previously [19]. Each food parameter was assigned a score of + 1 if it exhibited pro-inflammatory effects, −1 if it demonstrated anti-inflammatory properties, or 0 if it induced no significant changes in inflammation biomarkers. Essentially, a greater positive DII rating signifies a dietary pattern inclined toward pro-inflammation, whereas a lesser or negative rating indicates a diet leaning toward anti-inflammatory effects. Twenty-three foods/nutrients were used to calculate DIIs, which included energy, protein, total fat, carbohydrate, cholesterol, fiber, iron, niacin, riboflavin, thiamine, vitamin A, vitamin C, vitamin E, selenium, beta-carotene, zinc, magnesium, alcohol, garlic, ginger, onion, green/black tea, and isoflavones.

### Body measurement

All physical indicators were measured in accordance with the industry standard of the People’s Republic of China, namely, the “Anthropometric measurements method in health surveillance (WS/T424-2013)” [46]. A metal column height meter was used to measure the height. The maximum measurement distance is 2.0 m, and the accuracy is 0.1 cm. An Hd-390 (TANITA, Japan) electronic scale was used to measure body weight; the maximum weight was 150 kg, and the measurement accuracy was 0.1 kg. Blood pressure was measured with an electronic sphygmomanometer Omron-HBP1300, and the measurement accuracy was 1 mmHg.

### Biomarker evaluation

FBG was collected in the morning and measured via the glucose oxidase method. The diagnostic criteria for diabetes are based on the Guidelines for the Prevention and Treatment of Type 2 Diabetes Mellitus in China (2020 edition) [47]. DDM patients were defined as those who were diagnosed with diabetes by a regular hospital or who were taking hypoglycemic drugs to control their blood sugar. UDDM was defined as self-reported without diabetes but with a FBG ≥ 7.0 mmol/L. Overweight, obesity and central obesity were determined by BMI according to the guidelines for prevention and control of overweight and obesity in Chinese adults [48]. A BMI between 18.5 and 23.9 kg/m² was considered normal. Overweight was defined as a BMI of 24.0 to 27.9 kg/m², while a BMI of 28.0 kg/m² or greater indicated obesity. Consequently, 24 kg/m² served as the cutoff point for identifying overweight or obesity in this Chinese population. Central obesity was judged as a waist circumference ≥ 85 cm in men and ≥ 80 cm in women. Hypertension was defined according to the Chinese guidelines for hypertension prevention and treatment (2024 revision) [49] as having a systolic blood pressure (SBP) > 140 mmHg and/or a diastolic blood pressure (DBP) > 90 mmHg.

### Statistical analysis

For continuous variables, a t test or one-way ANOVA was performed for normally distributed data, whereas the Mann‒Whitney U test was used for nonnormally distributed data. The chi-square test was used for categorical variables. This cross-sectional study employed an unmatched design without individual-level matching between the case and control groups, and therefore utilized an unconditional logistic regression model. The potential confounders were selected based on directed acyclic graph of the relationship between DII, BMI, WtHR and diabetes (Figure **S1**).

We also employed restricted cubic spline (RCS) models [50, 51] to assess the linear or nonlinear associations between the DII, BMI, and WtHR and the risk of DM. The knots within the RCS models were automatically determined on the basis of the minimum Akaike information criterion (AIC) value.

In a recent study [52], the WQS model was used to provide a comprehensive explanation of the triglyceride glucose-body mass index by calculating the weights of FBG, triglyceride-glucose, and BMI. The WQS model has also been employed to investigate the associations among the planetary health diet index, individual nutrients, and asthma [53]. Therefore, we also apply the WQS model to elucidate the combined associations among DII, BMI, the WtHR, and DDM or UDDM.

SPSS 27.0 for Windows (SPSS Inc., Chicago, IL, USA) and R4.4.0 (R Development Core Team) were used for the statistical analyses. A two-sided *P value* < 0.05 indicated statistical significance.

## Results

### Basic characteristics of the study subjects

**Fig. 2:**
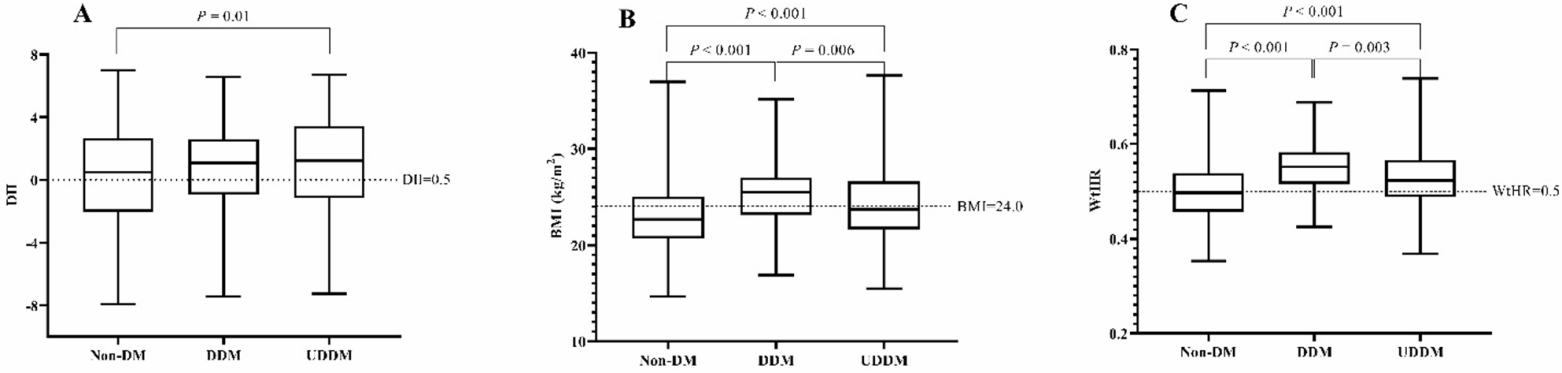
Comparison DII, BMI, and the WtHR by different diagnoses of diabetes based on one-way ANOVA. Comparison of the DII score according to Non-DM, DDM and UDDM (**A**); Comparison of the BMI according to Non-DM, DDM and UDDM (**B**); Comparison of the WtHR according to Non-DM, DDM and UDDM (**C**)

Among 3,687 eligible subjects, the prevalence of TDM was 6.6%, with 40.6% DDM and 59.4% UDDM cases. The DII scores of all the participants ranged from − 7.91 to 6.98, with a median (P_25_, P_75_) of 0.53 (−1.98, 2.71). The BMI of all the participants ranged from 14.65 to 37.63, with a median (P_25_, P_75_) of 22.83 (20.77, 25.22). The comparison of the basic characteristics of non-DM and DM patients is presented in Table 1. The characteristics of the participants according to quintiles of the DII are shown in Table S1. Compared with non-DM, the TDM subjects had higher FBG, DII, age, weight, BMI, WC, WtHR, SBP, and DBP levels. The TDM subjects were more likely to live in urban areas, have less physical activity, have a lower education level, have a higher BMI and WC, and have a higher incidence of overweight, central obesity and hypertension. Moreover, compared with DDM subjects, UDDM subjects had higher FBG, younger age, BMI, and WtHR. UDDM subjects were also more likely to be female, live in rural areas, and have less physical activity. As shown in Fig. 2 and Table S2, compared with the non-DM group, the UDDM group had higher DII scores. Compared with the non-DM group, both the DDM and UDDM groups had higher BMIs and WTRs.

**Table 1:** Comparison of the basic characteristics of participants with different diabetes diagnoses

Variables	Overall subjects (*N*=3687)	Different diabetes diagnoses					
		Non-DM (*N*=3443)	TDM (*N*=244)	*P*	TDM		*P*
					DDM (*N*=99)	UDDM (*N*=145)	
Continuous variable, median (IQR)							
FBG (mmol/L)	5.2 (4.8,5.7)	5.2 (4.8, 5.6)	7.9 (7.1, 9.7)	<0.001	6.8(5.7, 9.0)	8.2(7.4, 10.1)	<0.001
DII	0.53 (−1.98, 2.71)	0.48 (−2.03, 2.68)	1.21 (−1.06, 3.07)	0.004	1.07(−0.93, 2.61)	1.23(−1.13, 3.42)	0.580
Age (year)	50 (39, 60)	49 (38, 59)	57 (49, 63)	<0.001	61(53, 64)	55(45, 63)	0.002
Height (cm)	157.3 (152.0,163.2)	157.4 (152.0,163.2)	156.5 (150.9,163.2)	0.289	155.6(150.5, 162.1)	158(151.2, 164)	0.143
Weight (kg)	56.9 (50.4, 64.0)	56.6 (50.1, 63.5)	61 (53.9, 67.5)	<0.001	61.4(54.4, 68.7)	60.6(53.5, 66.8)	0.280
BMI	22.8 (20.8, 25.2)	22.7 (20.7, 25.1)	24.5 (22.5, 26.7)	<0.001	25.5(23.2, 27)	23.7(21.7, 26.6)	0.006
Waistline (cm)	79 (72, 85)	78 (72, 85)	85 (78, 90)	<0.001	86(80, 90)	83(77, 90)	0.055
WtHR	0.50 (0.46, 0.54)	0.50 (0.46, 0.54)	0.54 (0.50, 0.57)	<0.001	0.55(0.51, 0.58)	0.53(0.49, 0.57)	0.002
SBP (mmHg)	127 (115, 141)	127 (115, 141)	136 (124, 152)	<0.001	138(127, 153)	136(123, 152)	0.511
DBP (mmHg)	78 (71, 86)	78 (70, 86)	80 (75, 89)	<0.001	79(74, 86)	81(75, 91)	0.115
Categorical variable, *n* (%)							
Gender, *n* (%)				0.221			0.009
Male	1665(45.2)	1564(45.4)	101(41.4)		31(31.3)	70 (48.3)	
Female	2022(54.8)	1879(54.6)	143(58.6)		68(68.7)	75 (51.7)	
Region, *n* (%)				<0.001			<0.001
Urban	970(26.3)	872(25.3)	98(40.2)		53(53.5)	45(31.0)	
Rural	2717(73.7)	2571(74.7)	146(59.8)		46(46.5)	100(69.0)	
Education levels, *n* (%)				0.009			0.590
Primary and below	1552(42.1)	1427(41.5)	125(51.2)		50(50.5)	75(51.7)	
Junior high school	1361(36.9)	1289(37.4)	72(29.5)		27(27.3)	45(31.0)	
High School and above	774(21.0)	727(21.1)	47(19.3)		22(22.2)	25(17.2)	
Physical activity, *n* (%)				<0.001			<0.001
Light	1721(46.7)	1574(45.7)	147(60.3)		75(75.8)	72(49.7)	
Medium	1087(29.5)	1034(30.0)	53(21.7)		13(13.1)	40(27.6)	
Heavy	879(23.8)	835(24.3)	44(18.0)		11(11.1)	33(22.7)	
Occupation, *n* (%)				0.005			0.119
Office worker	1689(45.8)	1556(45.2)	133(54.5)		60(60.6)	73(50.3)	
Agriculture & farming	1998(54.2)	1887(54.8)	111(45.5)		39(39.4)	72(49.7)	
Smoking, *n* (%)				0.587			0.119
No	2803(76.0)	2614(75.9)	189(77.5)		82(82.8)	107(73.8)	
Yes	884(24.0)	829(24.1)	55(22.5)		17(17.2)	38(26.2)	
Drinking, *n* (%)				0.119			0.287
No	2635(71.5)	2450(71.2)	185(75.8)		79(79.8)	106(73.1)	
Yes	1052(28.5)	993(28.8)	59(24.2)		20(20.2)	39(26.9)	
BMI, *n* (%)				<0.001			0.011
Normal	2099(56.9)	1996(58.0)	103(42.2)		31(31.3)	72(49.7)	
Overweight	1056(28.6)	964(28.0)	92(37.7)		49(49.5)	43(29.7)	
Obesity	291(7.9)	254(7.3)	37(15.2)		15(15.2)	22(15.1)	
Emaciation	241(6.6)	229(6.7)	12(4.9)		4(4.0)	8(5.5)	
Central obesity, *n* (%)				<0.001			0.007
No	2291(62.1)	2197(63.8)	94(38.5)		28(28.3)	66(45.5)	
Yes	1396(37.9)	1246(36.2)	150(61.5)		71(71.7)	79(54.5)	
Hypertension, *n* (%)				<0.001			0.304
No	2530(68.6)	2402(69.8)	128(52.5)		48(48.5)	80(55.2)	
Yes	1157(31.4)	1041(30.2)	116(47.5)		51(51.5)	65(44.8)	
Family history of diabetes, *n* (%)				<0.001			<0.001
No	3553(96.4)	3332(96.8)	221(90.6)		80(80.8)	141(97.2)	
Yes	134(3.6)	111(3.2)	23(9.4)		19(19.2)	4(2.8)	

### The association between DII score and FBG

**Fig. 3:**
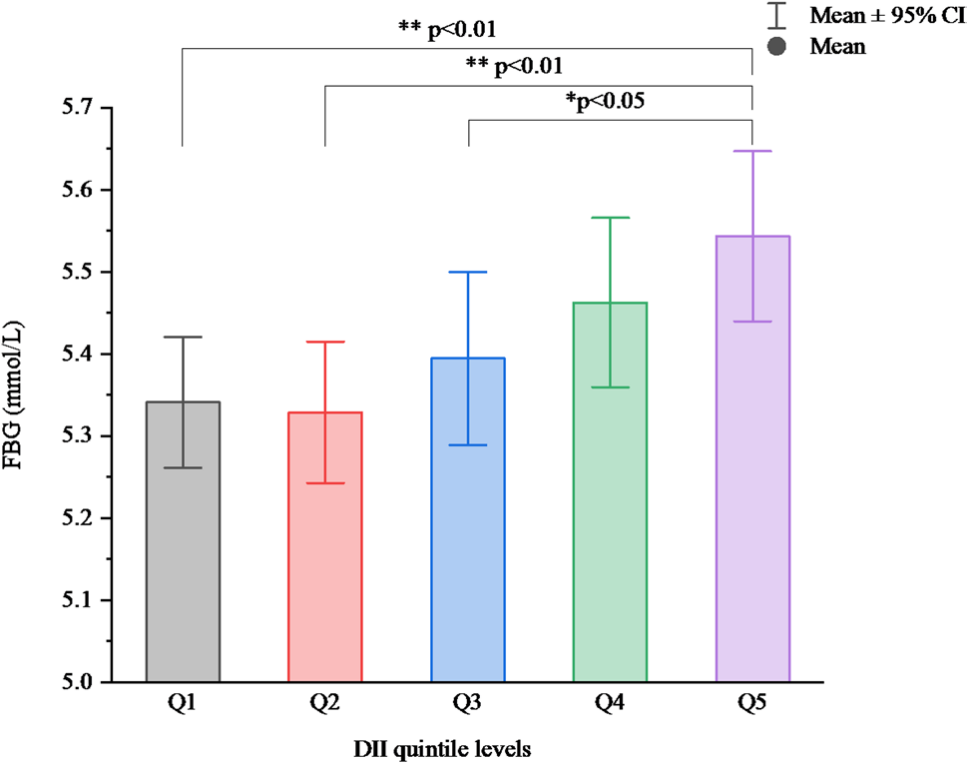
Comparison of FBG levels according to the DII quintile in the subjects

The standardized beta coefficients of the association between DII and FBG are shown in Table S3. The collinearity analysis revealed variance inflation factors (VIFs) of 1.004 for DII, 3.061 for BMI, and 3.059 for WtHR, all of which were below 5. These results indicate only a mild degree of collinearity, confirming the appropriateness of including these variables in the model. After controlling for confounders, the DII was significantly positively correlated with FBG. Moreover, the DII score tended to be associated with higher FPG (F = 3.334, *P* = 0.010; *P*-trend < 0.001). Participants in the highest quintile of DII score (Q5) have higher FPG than do those in the lowest level of DII (Q1) and those in the second quintile of DII score (Q3) (Fig. 3).

### Association between DII score and diabetes risk based on logistic regression analysis

The associations between DII quintile levels and the risk of TDM, DDM, and UDDM are shown in Table 2. The DII quintile was positively associated with an increased risk of both TDM and UDDM (*P*_− trend_ tests all ≤ 0.05). Compared with the lowest level of DII (Q1), the ORs (95% CIs) of TDM in the Q4 and Q5 quintiles were 1.66 (1.07, 2.57) and 1.84 (1.19, 2.86) for Model 2 and 1.65 (1.06, 2.56) and 1.88 (1.21, 2.92) for Model 3, respectively. For UDDM, individuals in the highest DII quintile (Q5) presented a significantly greater risk than did those in the lowest quintile (Q1), with ORs (95% CIs) of 1.94 (1.14, 3.32) in Model 2 and 1.98 (1.16, 3.40) in Model 3. Moreover, the DII quintile was not positively associated with DDM risk. The risk was significantly greater in Q4 than in Q1, with ORs (95% CIs) of 2.03 (1.03, 3.99) in Model 2 and 1.99 (1.01, 3.91) in Model 3.

**Table 2:** The association between DII quintile levels and DM risk

DM	DII quintile	Case (%)	OR (95% CI)		
			Model 1	Model 2	Model 3
TDM	Q1 (≤ −2.52)	38 (5.2)	1.00 (Reference)	1.00 (Reference)	1.00 (Reference)
	Q2 (−2.58, −0.43)	39 (5.3)	1.03 (0.65, 1.63)	1.08 (0.68, 1.73)	1.07 (0.67, 1.71)
	Q3 (−0.42, 1.38)	51 (6.9)	1.37 (0.89, 2.11)	1.54 (0.99, 2.41)	1.53 (0.98, 2.40)
	Q4 (1.39, 3.20)	57 (7.7)	1.54 (1.01, 2.36)	1.66 (1.07, 2.57)	1.65 (1.06, 2.56)
	Q5(≥ 3.21)	59(8.0)	1.60 (1.05, 2.44)	1.84 (1.19, 2.86)	1.88 (1.21, 2.92)
		*P*-trend	0.006	0.001	< 0.001
DDM	Q1 (≤ −2.52)	15 (2.0)	1.00 (Reference)	1.00 (Reference)	1.00 (Reference)
	Q2 (−2.58, −0.43)	15 (2.0)	1.00 (0.49, 2.06)	1.08 (0.51, 2.29)	1.06 (0.50, 2.25)
	Q3 (−0.42, 1.38)	22 (3.0)	1.48 (0.76, 2.87)	1.73 (0.86, 3.47)	1.70 (0.85, 3.42)
	Q4 (1.39, 3.20)	27 (3.7)	1.83 (0.97, 3.47)	2.03 (1.03, 3.99)	1.99 (1.01, 3.91)
	Q5(≥ 3.21)	20 (2.7)	1.34 (0.68, 2.64)	1.60 (0.78, 3.27)	1.62 (0.79, 3.32)
		*P*-trend	0.127	0.055	0.052
UDDM	Q1 (≤ −2.52)	23 (3.2)	1.00 (Reference)	1.00 (Reference)	1.00 (Reference)
	Q2 (−2.58, −0.43)	24 (3.3)	1.04 (0.58, 1.87)	1.10 (0.61, 1.98)	1.09 (0.61, 1.97)
	Q3 (−0.42, 1.38)	29 (3.9)	1.28 (0.73, 2.24)	1.41 (0.80, 2.48)	1.42 (0.81, 2.49)
	Q4 (1.39, 3.20)	30 (4.0)	1.34 (0.77, 2.33)	1.46 (0.83, 2.55)	1.46 (0.83, 2.57)
	Q5(≥ 3.21)	39 (5.3)	1.75 (1.03, 2.95)	1.94 (1.14, 3.32)	1.98 (1.16, 3.40)
		*P*-trend	0.022	0.008	0.006

Model 1: Crude model

Model 2: Adjusted for sex, region, age, education level, physical activity, occupation, smoking status, alcohol consumption status, hypertension status, family history of diabetes, and BMI

Model 3: Adjusted for gender, region, age, education level, physical activity, occupation, smoking, drinking, hypertension, family history of diabetes, and the WtHR

### The associations between DII, BMI, and the WtHR and diabetes risk based on RCS models

**Fig. 4:**
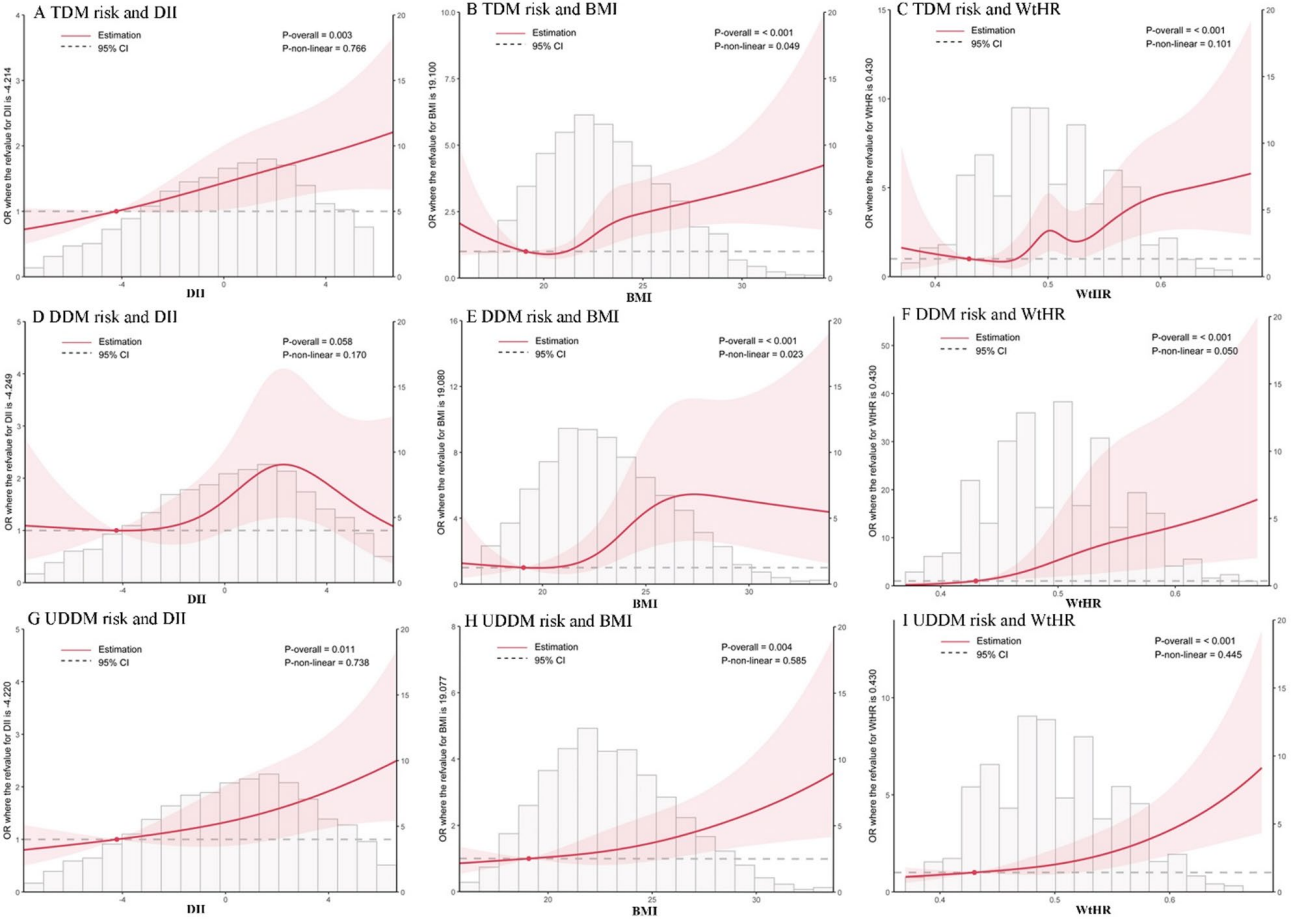
The RCS models for the associations between DII, BMI, and the WtHR and the risk of diabetes. The red solid lines, pink shaded areas, and gray dotted lines indicate ORs, CIs, and reference lines (OR = 1), respectively. The gray histogram indicates the fraction with different DII, BMI, and WtHR values. RCS models for the associations between total TDM risk and DII (**A**), BMI (**B**), and WtHR (**C**); RCS models for the associations between DDM risk and DII (**D**), BMI (**E**), and WtHR (**F**); and RCS models for the associations between UDDM risk and DII (**G**), BMI (**H**), and WtHR (**I**). Sex, region, age, education level, physical activity, occupation, smoking status, drinking status, hypertension status, and family history of diabetes were adjusted for in all RCS models. In the RCS models of DII and diabetes (**A**, **D**, and **G**), BMI was also adjusted. In the RCS models of BMI or the WtHR and diabetes status (**B**, **C**, **E**, **F**, **H** and **I**), the DII was also adjusted

As shown in Fig. 4, DII scores and the risks of TDM and UDDM were linear. (all *P*-overall values < 0.05). There was no association between the DII and DDM risk. BMI and the risk of TDM and DDM were nonlinear (all *P* values were < 0.05), whereas the linear relationships between BMI and UDDM risk (*P*-overall value < 0.001). Moreover, the associations between the WtHR and the risks of TDM, DDM and UDDM were all linear (all *P*-overall values < 0.001).

### The interaction effects of DII, BMI, and the WtHR on DM risk based on WQS models

**Fig. 5:**
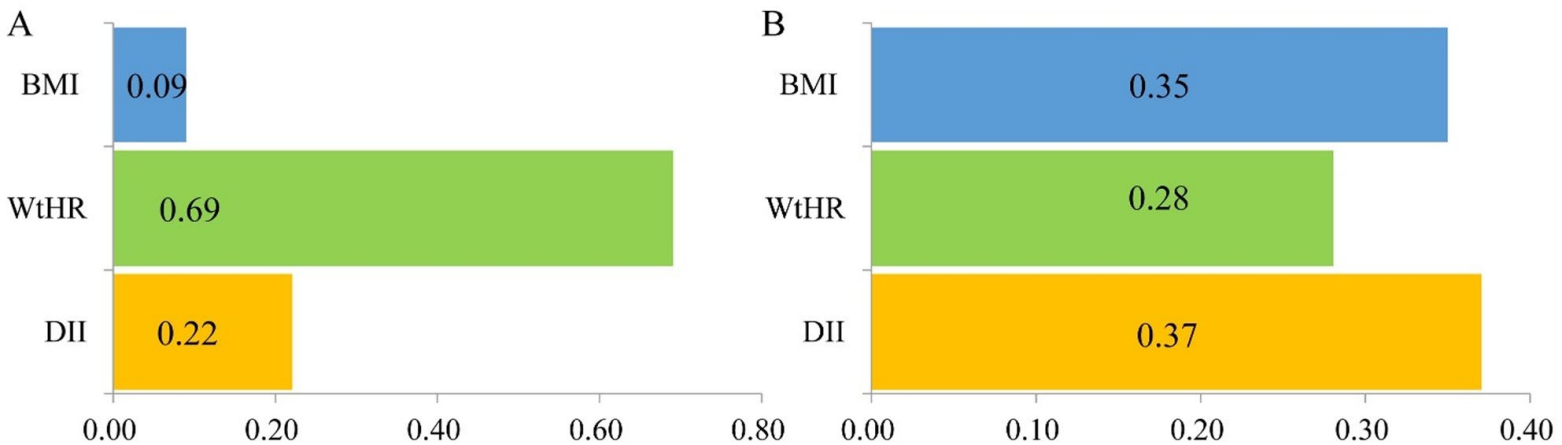
WQS model regression index weights for the risk of both DDM (**A**) and UDDM (**B**). The models were adjusted for sex, region, age, education level, physical activity, occupation, smoking status, drinking status, hypertension status, and family history of diabetes

In addition, the WQS index from the WQS regression was positively associated with the risk of both DDM and UDDM. Figure 5 revealed that DII, BMI, and the WtHR were positively associated with both DDM and UDDM. For DDM, the WtHR emerged as the most influential factor (weight = 0.69), followed by DII (weight = 0.22) and BMI (weight = 0.09). In the UDDM patients, DII was identified as the primary determinant (weight = 0.37), followed by BMI (weight = 0.35) and the WtHR (weight = 0.28).

## Discussion

### Prevalence comparison

The characteristics of the previous studies included in the comparison of the results from our study are shown in Table S4. Our study indicates that the TDM prevalence among the study population is 6.6%, with DDM and UDDM constituting 40.6% and 59.4% of the TDM cases, respectively. The UDDM prevalence in our study was higher than the adult diabetes patients in China, which was 49.7% in 2024 [7]. This finding highlights the concealed nature of UDDM and the subsequent public health risks it poses. In support of this, data from 2024 revealed that nearly half of all adults (aged 20 to 79 years) with diabetes were unaware of their condition, accounting for 42.8% or 251.7 million individuals [7]. The prevalence rate of type 2 diabetes in the Chinese population increased from 0.67% in 1980 to 11.2% in 2015 to 2017 [47]. The prevalence of diabetes in this study was lower than that reported in China [47], Xinjiang, China [24], Hubei Province, China [25], Jilin Province, China [33], the United States [16, 27], and Mexico [29]. This variation can be attributed to differences in study populations and research timelines across studies. Given that diabetes is a disease closely linked to diet and physical activity, substantial differences can arise among diverse populations [8, 12, 54, 55]. The relationship between DII and DM appears to vary significantly among different ethnic groups [24, 56]. Specifically, our study population was predominantly from Guangxi, an economically less developed region with a majority rural population characterized by individuals with normal weight and engaging in moderate to heavy physical activity.

### Association between DII and DDM

Compared with the participants in Q1 (the lowest pro-inflammatory category), the participants in Q4 of DDM and the participants in Q5 of UDDM (the highest pro-inflammatory category) showed higher odds of diabetes mellitus prevalence. On the one hand, previous studies [16, 24–29] support positive correlations of the DII with the TDM and DDM. These studies, which used cross-sectional and cohort designs, involved both males and females. In this study, a higher DII exhibited a significant link to the increased risk of TDM, DDM, and UDDM after adjustment for potential confounders. Compared with the findings of previous studies conducted in the general adult population [16, 24–29, 31, 32], the findings of our study differed from those of two studies [32], which reported nonsignificant associations between DII and DM risk. These discordant findings may partially stem from the variations in the study population. The study population was from the Tehran Lipid and Glucose Study, which was a cross-sectional study [32]. The majority of participants consumed anti-inflammatory diets, and the DII score in the present study [32] was lower than that in our study and those with positive results [16, 24–29]. In contrast to the aforementioned studies, our research employed a 3-day 24-hour dietary recall method combined with a weighing method for dietary data collection. While the previous studies utilized FFQs to calculate the DII, both dietary assessment methods can be applied for DII computation and association analysis. However, our approach demonstrates distinct advantages: not only does the 3-day 24-hour dietary recall provide comprehensive food consumption data, but the supplementary weighing method also captures precise condiment intake information. This dual-method strategy enables more complete dietary intake assessment, thereby enhancing the accuracy and reliability of our findings. Furthermore, a US cohort study [31] revealed no significant association between DII scores and type 2 diabetes incidence in multivariable models, although point estimates consistently increased across increasing DII quartiles. The study [31] enrolled healthy, motivated participants in the Aerobics Center Longitudinal Study, with a low proportion of obese individuals. The sample was predominantly well-educated White males, limiting generalizability. Our research subjects were the general population (aged 18–69 years), and they were characterized by being from rural areas and having a lower level of education. The inconsistency in the characteristics of the population was another reason for the inconsistency in the research results.

### Association between DII and UDDM

On the other hand, studies on the association between DII and UDDM are limited globally. Our study represents an inaugural comparison of the DII between DDM and UDDM in the general Chinese population. In our cross-sectional analysis, individuals with UDDM in the highest DII quintile (Q5) exhibited a statistically significant association with elevated risk relative to the lowest quintile (Q1). Moreover, the DII quintile was not positively associated with DDM risk. Moreover, RCS models revealed that the DII was linearly associated with UDDM risk but not with DDM risk in a statistically significant linear or nonlinear dose‒response manner. Compared with participants with diabetes, those with undiagnosed diabetes had higher DII scores in the US general population [26]. Another study [16] reported that a higher DII increased the odds of undiagnosed diabetes, with an OR (95% CI) of 1.31 (1.16, 1.50). These findings align with our results, whereas these studies failed to identify a dose‒response relationship. The present study employs a comprehensive approach that includes multivariate logistic regression analysis and the RCS model to explore the association between DII and UUDM. The mechanism of diet and DM is that a proinflammatory diet can contribute to an increase in chronic inflammation in the body [57]. A high DII, a proinflammatory diet promoting low-grade chronic inflammation, thereby reduces insulin sensitivity [17].

### Associations between BMI/WtHR and UDDM

Our results revealed that the relationship between BMI and DDM risk was nonlinear but linear with UDDM risk. Significant positive linear response associations were also found between the WtHR and the risks of both DDMs and UDDM. Although BMI and the WtHR can both be used as indicators reflecting body type, the WtHR reflects the degree of fat accumulation in the waist and abdomen of the human body [36]. A higher WtHR indicates a larger waist circumference and more visceral fat accumulation. Rahimlou et al. [39] and Namazi et al. [38] reported that higher WtHR values were significantly correlated with increased diabetes risk. In a cross-sectional study in Jilin, China [33], the WtHR was more strongly associated with diabetes than BMI among participants ≥ 40 years of age, especially females. Furthermore, two studies [37, 58] have suggested that the WtHR serves as an effective metric for screening for central obesity. A study conducted in China [59] demonstrated that the association between the WtHR and type 2 diabetes is stronger than that between BMI and type 2 diabetes. In this study, we employed RCS models to investigate the relationship between the WtHR and DDM or UDDM. Our findings revealed that the optimal cutoff point for the WtHR in individuals with UDDM was 0.43 (Table S5), which is lower than the threshold of 0.5 proposed by Anurag Bajpai [37]. These insights regarding the WtHR and its association with DDM or UDDM will enable the development of earlier intervention strategies for populations at risk.

### Contribution of DII/BMI/WtHR to DDM/UDDM

The WQS regression models revealed that the WtHR was the most influential factor (weight = 0.69), with BMI contributing minimally (weight = 0.09) in participants with DDM. For participants with UDDM, DII emerged as the primary determinant, outweighing BMI (weight = 0.37 vs. 0.35) and the WtHR (weight = 0.28). Participants with UDDM, who are free of known diabetes, maintain their usual dietary habits, ensuring that the dietary information collected concurrently with blood samples accurately represents their typical intake [16]. Our findings reveal significant variations in the contributions of the DII, BMI, and WtHR across different stages of diabetes. For participants with DDM, following a definitive diagnosis, the initiation of glucose-lowering medications, along with dietary modifications and weight loss, results in diminished influences of DII and BMI, whereas the role of the WtHR becomes more prominent. This is attributable to the fact that the WtHR serves as a more representative indicator of visceral fat accumulation than BMI does [36]. BMI, which is traditionally used to diagnose obesity, inadequately reflects body fat percentage and visceral fat distribution, which are key factors in defining obesity and its associated chronic disease risk [36]. Despite reductions in BMI among DDM patients, the WtHR may still exceed healthy ranges, explaining the nonlinear association observed between BMI and DDM risk in RCS models. Conversely, among UDDM individuals, who are unaware of their glycemic status and consequently do not undergo significant alterations in dietary or physical activity habits, the influence of DII is paramount. In light of the fact that neither the current Guidelines for the Diagnosis and Treatment of Obesity (2024) [40] nor the Chinese Guidelines for the Prevention and Treatment of Diabetes Mellitus (2024) [41] [have incorporated WtHR as an assessment parameter, we propose that WtHR should be included as a key reference metric in guidelines for the prevention and management of overweight/obesity and diabetes mellitus. For the health management of DDM, emphasis should be placed on WtHR monitoring alongside BMI. Given the contribution of DII to UDDM, in the health education knowledge for high-risk groups of diabetes, adding guidance on stress-resistant diets and introducing anti-inflammatory dietary patterns as a recommended dietary pattern to high-risk groups may play a positive role in the prevention of diabetes.

Healthier diets may reduce risk of diabetes both by reducing weight gain and other mechanisms such as reducing inflammation [28, 60]. Another study [31] involving a population of mostly healthy weight-educated males reported no associations between obesity, DII, and type 2 diabetes; however, this limits the generalizability of the findings. Visceral fat accumulation poses a greater risk of metabolic disorders [58], which may be one of the reasons why we observed a stronger impact of the WtHR on the relationship between DII and type 2 diabetes than BMI in our study. Our study results also suggest that the roles of BMI and the WtHR in the progression of diabetes cannot be ignored. Among individuals with DDM, in addition to the DII, special attention should be given to the WtHR. For those with UDDM, both the DII and obesity factors, including BMI and the WtHR, should be managed and controlled. These findings further indicate that different management and control measures should be adopted for diabetes patients at various stages of the disease.

### Strengths and limitations

This study represents the first comprehensive analysis utilizing traditional logistic regression analysis, RCS, and WQS modeling to investigate the associations between the DII, BMI, and WtHR and both diagnosed and undiagnosed diabetes mellitus in the general Chinese population. Our findings provide valuable insights into the characteristics of the general adult population with undiagnosed diabetes and highlight the urgent necessity of implementing early intervention strategies for different diabetes diagnoses on the basis of diet and obesity. Compared with other methods [9, 28, 31], our dietary survey method, which combines 3-day 24-hour dietary recall (2 weekdays, 1 weekend) with the weighing of household seasonings, allows for more accurate allocation of seasoning intake to participants, yielding a comprehensive dataset reflecting real-life dietary habits.

There are several limitations in this study. First, owing to the cross-sectional nature of the study design, temporal or causal associations cannot be established. Notably, the absence of sampling weights in the original datasets prevented us from accounting for clustering and design effects in the statistical analysis. This limitation may result in underestimated variances and excessively narrow 95% confidence intervals (CIs) for the odds ratios (ORs), which could inflate the statistical significance of the observed associations. Nevertheless, our findings offer valuable insights for exploring potential relationships, and future studies with complete sampling weight data are recommended to verify these results. Second, out of the 45 parameters assessed, only 23 were derived from the 3-day 24-hour dietary recall, a limitation attributed to the questionnaire design and the distinctive dietary patterns in Guangxi, China. The number of nutrients and food items utilized in the computation of the DII in the current study is lower than that in previous investigations [16, 24–29, 31, 32], where the range typically spans from 26 to 29 parameters. Notably, the comprehensive DII scoring framework encompasses 45 food parameters; nevertheless, evidence [61, 62] has shown that the DII retains its predictive validity even when it is calculated using fewer than 30 food parameters. Moreover, a study [63] has shown that a DII based on 17 nutrients can also predict various inflammatory markers in study participants. Third, this study was conducted in Guangxi, a representative city in southern China. The sample size is relatively small. Participants with missing covariate data were excluded. Little’s missing completely at random (MCAR) test was used to evaluate the pattern of missing covariate data, yielding a significant result (*χ*²=296.001, *df* = 12, *P* < 0.001), which indicates that the missing data were not completely random. Given the low overall proportion of missing data (145/3832 = 3.78%), we considered the risk of bias from a complete case analysis to be acceptable. Specifically, the participants were predominantly from rural areas (73.7%), had relatively low educational attainment (42.0% with primary school education or below, and 36.9% with junior high school education), and were primarily of Han ethnicity (71.4%). Additionally, distinct dietary cultures and structures in this region differ from those in other areas. These potentially limit the generalizability of our findings. Although we carefully accounted for relevant confounding factors, several important variables remained unaddressed in our analysis. Emerging research demonstrates that sleep disorders [64], particularly obstructive sleep apnoea and insomnia, impair insulin sensitivity and promote resistance, heightening type 2 diabetes mellitus (T2DM) risk. Mental health indicators also show robust associations with T2DM [65] where moderate-to-severe distress, life satisfaction scores ≤ 3, clinical depression, and neuroticism scores ≥ 3 independently predict diabetes incidence [66]. Our study did not adjust for these parameters. Furthermore, while T2DM exhibits substantial heritability [67], we omitted genetic polymorphism analyses. These limitations may affect the precision of our findings.

## Conclusions

In summary, our study revealed that high DIIs significantly increase the risk of DDM and UDDM. Our findings indicate that a diet exhibiting high proinflammatory potential is linked to an elevated risk of developing both DDM and UDDM. Adiposity factors, assessed in this study via BMI and the WtHR, also emerged as significant contributors to the onset of DDM and UDDM, albeit with varying degrees of influence. The results enhance our understanding of the underlying mechanisms through which diet and obesity contribute to the manifestation of DDM and UDDM. More studies, especially large-scale population studies, are needed to verify these findings.

## Supplementary Information

Supplementary Material 1.

## Data Availability

The dataset generated and used for this study is not publicly available but is available from the corresponding author on reasonable request.

## Abbreviations

BMI: Body Mass Index
CACDNS: the China Adult Chronic Disease and Nutrition Surveillance
CNNHS: the China National Nutrition and Health Survey
DDM: Diagnosed Diabetes Mellitus
DII: Dietary Inflammation Index
DM: Diabetes Mellitus
FBG: Fasting Blood Glucose
RCS: Restricted Cubic Spline
TDM: Total Diabetes Mellitus
UDDM: Undiagnosed Diabetes Mellitus
WQS: Weighted Quantile Sum
WtHR: Waist-to-Height Ratio
