# Supplementary material for: Associations between the dietary inflammatory index, body mass index, and waist-to-height ratio and diagnosed and undiagnosed diabetes mellitus in adults in Guangxi, China
Source: BMC Public Health. 2025 Dec 26;26:365. doi: 10.1186/s12889-025-25942-9 (PMC12849686; doi:10.1186/s12889-025-25942-9)
Supplement: Supplementary file 1 — Supplementary Material 1. [file 12889_2025_25942_MOESM1_ESM.docx]

**Supplementary Materials**

**Associations between the dietary inflammatory index, body mass index, and waist-to-height ratio and diagnosed and undiagnosed diabetes mellitus in adults in Guangxi, China**

Zhongyou Li ^1, #^, Yan Li ^2, #^, Hai Li ^1, #^, Zhifeng Fang ^2^, Yuzhu Chen ^2^, Xiaopeng Li ^2^, Qiulan Qing^2^, Weiwen Zhou ^2, *^, Jiongli Huang ^1, **^

^1^ *Department of Preventive Medicine, School of Public Health and Management, Guangxi University of Chinese Medicine, Nanning, 530200, China*

^2^ *Guangxi Zhuang Autonomous region Center for Disease Control and Prevention，Nanning 530028，China*

* Correspondence: 381011550@qq.com;

** Correspondence: [huangjiongli123@163.com](mailto:huangjiongli123@163.com);

^#^ These authors have contributed equally to this article.

**Tables**: 5

**Figure:** 1

**Pages**: 8

**Tables**

**Table S1** Characteristics of participants according to quintiles of the DII

**Table S2** Characteristics of DII, BMI, and WtHR in the development of diabetes

**Table S3** Standardized Beta coefficients of the association between DII and FBG

**Table S4** Characteristics of the studies included in the comparison of results from our study

**Table S5** Characteristics of different DII, BMI, and WtHR groups in the development of diabetes

**Figure**

**Figure S1** Directed acyclic graph of the relationship between DII, BMI, WtHR and diabetes.

Table S1 Characteristics of participants according to quintiles of the DII

| Variables | DII quintiles | | | | | *P* |
| --- | --- | --- | --- | --- | --- | --- |
|  | Q1 (n=737) | Q2 (n=737) | Q3 (n=738) | Q4 (n=737) | Q5 (n=738) |  |
| FBG (mmol/L), mean±SD ^a^ | 5.36±1.17 | 5.35±1.19 | 5.35±1.38 | 5.48±1.47 | 5.53±1.47 | 0.013 |
| Diabetes, n (%) ^b^ |  |  |  |  |  |  |
| Non-DM | 732 (94.4) | 743 (94.6) | 736 (93.6) | 725 (92.0) | 726 (92.2) | 0.196 |
| TDM | 44 (5.7) | 42 (5.4) | 50 (6.4) | 63 (8.0) | 61 (7.8) |  |
| DDM | 19 (2.4) | 13 (1.7) | 24 (3.1) | 28 (3.6) | 21 (2.7) |  |
| UDDM | 25 (3.2) | 29 (3.7) | 26 (3.3) | 35 (4.4) | 40 (5.1) |  |
| BMI (kg/m^2^), Median (IQR) ^a^ | 23.3 (21.0, 25.5) | 23.0 (20.8, 25.3) | 22.6 (20.8, 25.0) | 22.7 (20.6, 25.3) | 22.7 (20.8, 25.1) | 0.018 |
| BMI, n (%) ^b^ |  |  |  |  |  |  |
| Normal | 425 (54.6) | 449 (57.2) | 458 (58.3) | 445 (56.5) | 454 (57.7) | 0.053 |
| Overweight | 248 (32.0) | 221 (28.2) | 213 (27.1) | 214 (27.2) | 228 (29.0) |  |
| Obesity | 68 (8.9) | 68 (8.7) | 60 (7.6) | 71 (9.0) | 45 (5.7) |  |
| Emaciation | 35 (4.5) | 47 (6.0) | 55 (7.0) | 58 (7.4) | 60 (7.6) |  |
| WC (cm), Median (IQR) ^a^ | 80.4 (74.0, 87.0) | 79.0 (73.0, 85.3) | 78.0 (72.0, 85.3) | 78.0 (71.0, 86.0) | 78.0 (72.0, 84.0) | <0.001 |
| Central obesity, n (%) ^b^ |  |  |  |  |  |  |
| No | 430 (55.3) | 491 (62.5) | 498 (63.4) | 488 (61.9) | 515 (65.4) | 0.001 |
| Yes | 346 (44.7) | 294 (37.5) | 288 (36.6) | 300 (38.1) | 272 (34.6) |  |
| SBP (mmHg), Median (IQR)^a^ | 128 (117, 142) | 126 (115, 140) | 127 (114, 141) | 127 (117, 141) | 128 (116, 142) | 0.153 |
| DBP(mmHg), Median (IQR) ^a^ | 79 (71, 87) | 79 (71, 85) | 77 (70, 85) | 79 (71, 87) | 79 (70, 87) | 0.072 |
| Hypertension, n (%) ^b^ |  |  |  |  |  |  |
| No | 522 (67.3) | 559 (71.2) | 553 (70.4) | 531 (67.4) | 530 (67.3) | 0.236 |
| Yes | 254 (32.7) | 226 (28.8) | 233 (29.6) | 257 (32.6) | 257 (32.7) |  |
| Gender, n (%) ^b^ |  |  |  |  |  |  |
| Male | 420 (54.1) | 372 (47.4) | 352 (44.8) | 319 (40.5) | 335 (42.6) | <0.001 |
| Female | 356 (45.9) | 413 (52.6) | 434 (55.2) | 469 (59.5) | 452 (57.4) |  |
| Region, n (%) ^b^ |  |  |  |  |  |  |
| Urban | 255 (32.8) | 215 (27.4) | 218 (27.7) | 193 (24.5) | 168 (21.3) | <0.001 |
| Rural | 521 (67.2) | 570(72.6) | 568(72.3) | 595(75.5) | 619(78.7) |  |
| Education levels, n (%) ^b^ |  |  |  |  |  |  |
| Primary and below | 267 (34.4) | 308 (39.2) | 323(41.1) | 350(44.4) | 378(48.0) | <0.001 |
| Junior high school | 316 (40.7) | 313(39.9) | 269(34.2) | 273(34.6) | 298(37.9) |  |
| High School and above | 193 (24.9) | 164(20.9) | 194(24.7) | 165(20.9) | 111(14.1) |  |
| Physical activity, n (%) ^b^ |  |  |  |  |  |  |
| Light | 389 (50.1) | 350(44.6) | 352(44.8) | 373(47.3) | 378(48.0) | 0.01 |
| Medium | 195 (25.2) | 237(30.2) | 244(31.0) | 212(26.9) | 252(32.0) |  |
| Heavy | 192 (24.7) | 198(25.2) | 190(24.2) | 203(25.8) | 157(19.9) |  |
| Occupation, n (%) ^b^ |  |  |  |  |  |  |
| Office worker | 440 (56.7) | 356(45.4) | 363(46.2) | 347(44.0) | 313(39.8) | <0.001 |
| Agriculture & farming | 336 (43.3) | 429(54.6) | 423(53.8) | 441(56.0) | 474(60.2) |  |
| Smoking, n (%) ^b^ |  |  |  |  |  |  |
| No | 564 (72.6) | 587(74.8) | 604(76.8) | 614(77.9) | 598(76) | 0.133 |
| Yes | 212 (27.4) | 198(25.2) | 182(23.2) | 174(22.1) | 189(24) |  |
| Drinking, n (%) ^b^ |  |  |  |  |  |  |
| No | 518 (66.7) | 544(69.3) | 564(71.8) | 575(73.0) | 585(74.3) | 0.007 |
| Yes | 258 (33.3) | 241(30.7) | 222(28.2) | 213(27.0) | 202(25.7) |  |
| Family history of diabetes, n (%) ^b^ |  |  |  |  |  |  |
| No | 752 (96.9) | 755(96.2) | 766(97.5) | 749(95.1) | 760(96.6) | 0.113 |
| Yes | 24 (3.1) | 30(3.8) | 20(2.5) | 39(4.9) | 27(3.4) |  |

DII, dietary inflammation index; FBG, fasting blood glucose. Q1= first quintile, Q2= second quintile, Q3= third quintile, Q4=fourth quintile, and Q5=fifth quintile, respectively.

Table S2 Characteristics of DII, BMI, and WtHR in the development of diabetes

| Variables | | Non-DM (n=3443) | DDM (n=99) | UDDM (n=145) |
| --- | --- | --- | --- | --- |
| DII | Mean（SD） | 0.25±3.23 | 0.70±3.00 | 0.96±3.23 |
|  | Median（IQR） | 0.48 (-2.03,2.68) | 1.07 (-0.93,2.61) | 1.23 (-1.13,3.42) |
|  | Range | -7.91, 6.99 | -7.42, 6.57 | -7.24, 6.73 |
| BMI (kg/m^2^) | Mean（SD） | 23±3.2 | 25.2±3.1 | 24±3.6 |
|  | Median（IQR） | 22.7 (20.7,25.1) | 25.5 (23.2,27) | 23.7 (21.7,26.6) |
|  | Range | 14.65, 36.98 | 16.89, 35.16 | 15.48, 37.63 |
| WtHR | Mean（SD） | 0.50±0.06 | 0.55±0.05 | 0.53±0.06 |
|  | Median（IQR） | 0.5 (0.46,0.54) | 0.55 (0.51,0.58) | 0.52 (0.49,0.57) |
|  | Range | 0.35, 0.71 | 0.43, 0.69 | 0.37, 0.74 |

Table S3 Standardized Beta coefficients of the association between DII and FBG

| Models | *P*-value | Standardized Beta | 95%CI for Beta |
| --- | --- | --- | --- |
| Crude model | 0.005 | 0.057 | 0.023-0.084 |
| Model 1 ^a^ | 0.001 | 0.059 | 0.026-0.086 |
| Model 2 ^b^ | 0.001 | 0.062 | 0.029-0.089 |
| Model 3 ^c^ | ＜0.001 | 0.064 | 0.030-0.090 |

DII, dietary inflammation index; FBG, fasting blood glucose; BMI, body mass index; WtHR, waist to height ratio.

^a^ Model 1: Adjusting for gender, region, age, education levels, physical activity, occupation, smoking, drinking, hypertension and family history of diabetes.

^b^ Model 2: Adjusting factors in model 1 plus BMI.

^c^ Model 3: Adjusting factors in model 1 plus WtHR.

Table S4 Characteristics of the studies included in the comparison of results from our study

| **Reference** | **Study year** | **Study design** | **Study Area** | **Subjects** | **Sample size** | **Age (years)** | **Prevalence of DM** | **Dietary assessment methods** | **DII calculation** | **DII scores** | **DII classification** | **Comparison** |
| --- | --- | --- | --- | --- | --- | --- | --- | --- | --- | --- | --- | --- |
| Our study | 2012-2015 | Cross-sectional | Guangxi, China | General population | **3687** | **18-69** | DM 6.63%  DDM 2.69%  UDDM 3.93% | A 3-day, 24-hour (3d 24h) dietary survey combined with the weighing method | 23 food parameters | DM Range −7.91 to 6.98 Median (IQR) 0.53 (-1.98, 2.71)  DDM 1.07(-0.93,2.61)  UDDM 1.23(-1.13,3.42) | Quintiles | BMI was adjusted:  Q5 vs. Q1 for DDM  OR(95%CI)= 2.03 (1.03, 3.99)  Q5 vs. Q1 for UDDM OR(95%CI)= 1.94 (1.14, 3.32)  WtHR was adjusted:  Q5 vs. Q1 for DDM  OR(95%CI)= 1.99 (1.01, 3.91)  Q5 vs. Q1 for UDDM OR(95%CI)= 1.98 (1.16, 3.40) |
| [1] | 2019 | Cross-sectional | Xinjiang, China | General population | 5,105 | 35-74 | T2DM 8.2% | A FFQs consisting of 127 food items | 27 food parameters | Mean 0.81 | Quintiles | Q5 vs. Q1 OR(95%CI)= 3.27(2.38,4.50) |
| [2] | 2013-2016 | Cohort | Hubei, China | Pregnant women | 2,639 | ＞18 | GDM 13.1% | A semiquantitative FFQs consisted of 61 food items | 26 food parameters | Mean 0.55  Range -4.45 to +3.15 | Tertiles | Tertile3 vs. Tertile1 OR(95%CI)= 1.43(1.05,1.95) |
| [3] | 1990-1994(2003) | Cohort | Melbourne | General populations | 39,185 | 40-69 | / | A 121-item self-administered FFQs | 29 food parameters | Median (IQR) -1.0 (-2.2, 0.4) | Quintile | Q5 vs. Q1 for T2DM  IRR (95%CI) = 1.49 (1.30,1.72) |
| [4] | 2007-2018 | Cohort | US | General populations | 30,442 | ≥20 | DM 14.47%  UDDM 4.54% | 24-hour dietary recall | 28 food parameters | Non-DM 1.37(1.30-1.43)  UDDM 1.60(1.46-.73)  DM 1.67(1.56-1.77) | Low (−5.28,0.81)  Medium (0.81, 2.65)  High (2.65, 5.47) | / |
| [5] | 2007-2016 | Cross-sectional | US | General populations | 7,926 | / | DM 8.71% | Two 24-hour dietary recalls | 31 food parameters | Mean: −0.14  Range: −5.83-+5.32 | Tertiles | Tertile3 vs. Tertile1 OR(95%CI) for prediabetes = 1.40 (1.17, 1.69) Tertile3 vs. Tertile1 OR(95%CI) for Insulin Resistance = 1.79 (1.49, 2.14) |
| [6] | 2013-2014 | Cross-sectional | US | General populations | 4,434 | ≥20(Mean:49.4) | DM 14.1%  Prediabetes 26.5% | Two 24-hour dietary recalls | 28 food parameters | Mean: −0.65  With diabetes 0.79  Without diabetes 0.50  Range: −3.14-+9.05 | Continuous value | OR(95%CI) for diabetes = 1.13 (1.02, 1.24)  OR(95%CI) for HgbA1c = 1.43 (1.21, 1.68) |
| [7] | 2015 | Cross-sectional | Mexico | General populations | 1,174 | 20-69 | 13.6% | A validated semi-quantitative FFQs consisted of 140 foods. | 27 food parameters | Mean: −0.68  Range: −5.49 to +4.12 | Quintile | Q5 vs. Q1 for T2DM  OR (95%CI) = 3.02 (1.39,6.58) |
| [8] | 1993 - 2014 | Cohort | French | Women | 70,991 | Mean at baseline  53 | Incident T2DM 4.6% | A validated  208-item semi-quantitative dietary questionnaire | 32 food parameters | Mean −0.06 Range −13.42 to 24.8  Median −0.42 | Continuous variable | Higher DII scores were associated with a lower risk of T2DM. |
| [9] | 2009–2011 | Cross-sectional | Iran | General population | 2,975 | Mean 45 | / | A validated and reliable semi- FFQs | 37 food parameters | Range -5.82 to +5.23 | Continuous value and quartiles | No significant associations were observed between DII and risk of IFG, IGT, T2DM, and insulin resistance. DII had a positive weak association only with 2h-PG. |
| [10] | 1987-1999 | Cohort | US | Adult men | 6,016 | 20–84 | 336/6016=5.52% | A 3-day diet record | 26 food parameters | Mean -0.74  Range -4.31 to 3.66 | Quartile | DII scores were not significantly associated with T2DM incidence, but point estimates were consistently elevated across increasing DII quartiles. |

Table S5 Characteristics of different DII, BMI, and WtHR groups in the development of diabetes

| Variables | | Non-DM (n=3443) | DDM (n=99) | UDDM (n=145) |
| --- | --- | --- | --- | --- |
| DII | < 0 | 1550 (45.0) | 33 (33.3) | 53 (36.6) |
|  | ≥0 | 1893 (55.0) | 66 (66.7) | 92 (63.4) |
| BMI (kg/m^2^) | <24 | 2225 (64.6) | 35 (35.4) | 80 (55.2) |
|  | ≥24 | 1218(35.4) | 64 (64.6) | 65 (44.8) |
| WtHR | <0.5 | 1784 (51.8) | 16 (16.2) | 48 (33.1) |
|  | ≥0.5 | 1659 (48.2) | 83 (83.8) | 97 (66.9) |
|  | <0.43 | 388 (11.3) | 2 (2.0) | 8 (8.1) |
|  | ≥0.43 | 3055 (88.7) | 97 (98.0) | 91 (91.9) |


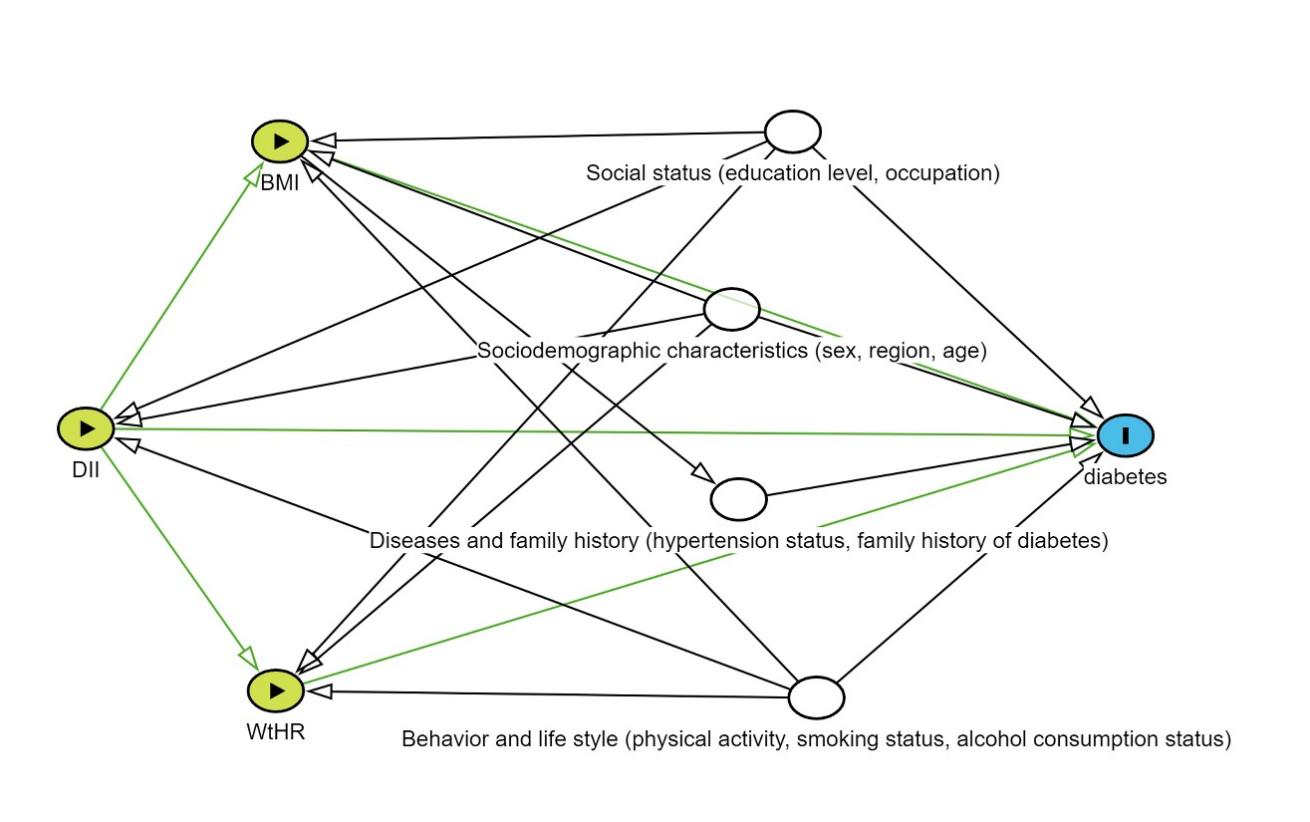


**Figure S1** Directed acyclic graph of the relationship between DII, BMI, WtHR and diabetes. The directed acyclic graphs in our study include sociodemographic characteristics (sex, region, age), Diseases and family history (hypertension status, family history of diabetes), and behavior and life style (physical activity, smoking status, alcohol consumption status).

**Reference**

1. Tang L, Shivappa N, Hebert JR, Lee AH, Xu F, Binns CW: **Dietary inflammatory index and risk of oesophageal cancer in Xinjiang Uyghur Autonomous Region, China**. *British Journal of Nutrition* 2018, **119**(9):1068-1075.

2. Zhang Z, Wu Y, Zhong C, Zhou X, Liu C, Li Q, Chen R, Gao Q, Li X, Zhang H *et al*: **Association between dietary inflammatory index and gestational diabetes mellitus risk in a prospective birth cohort study**. *Nutrition* 2021, **87-88**.

3. Hodge AM, Karim MN, Hebert JR, Shivappa N, de Courten B: **Association between Diet Quality Indices and Incidence of Type 2 Diabetes in the Melbourne Collaborative Cohort Study**. *Nutrients* 2021, **13**(11):4162.

4. Yuan S, He J, Wu S, Zhang R, Qiao Z, Bian X, Wang H, Dou K: **Trends in dietary patterns over the last decade and their association with long-term mortality in general US populations with undiagnosed and diagnosed diabetes**. *Nutrition and Diabetes* 2023, **13**(1):5.

5. Shu Y, Wu X, Wang J, Ma X, Li H, Xiang Y: **Associations of Dietary Inflammatory Index With Prediabetes and Insulin Resistance**. *Front Endocrinol (Lausanne)* 2022, **13**:820932.

6. King DE, Xiang J: **The Dietary Inflammatory Index Is Associated With Diabetes Severity**. *J Am Board Fam Med* 2019, **32**(6):801-806.

7. Denova-Gutierrez E, Munoz-Aguirre P, Shivappa N, Hebert JR, Tolentino-Mayo L, Batis C, Barquera S: **Dietary Inflammatory Index and Type 2 Diabetes Mellitus in Adults: The Diabetes Mellitus Survey of Mexico City**. *Nutrients* 2018, **10**(4):385.

8. Laouali N, Mancini FR, Hajji-Louati M, El Fatouhi D, Balkau B, Boutron-Ruault MC, Bonnet F, Fagherazzi G: **Dietary inflammatory index and type 2 diabetes risk in a prospective cohort of 70,991 women followed for 20 years: the mediating role of BMI**. *Diabetologia* 2019, **62**(12):2222-2232.

9. Moslehi N, Ehsani B, Mirmiran P, Shivappa N, Tohidi M, Hebert JR, Azizi F: **Inflammatory Properties of Diet and Glucose-Insulin Homeostasis in a Cohort of Iranian Adults**. *Nutrients* 2016, **8**(11):735.

10. Guinter MA, Merchant AT, Tabung FK, Wirth MD, Shivappa N, Hurley TG, Hebert JR, Sui X, Blair SN, Steck SE: **Adiposity does not modify the effect of the dietary inflammatory potential on type 2 diabetes incidence among a prospective cohort of men**. *J Nutr Intermed Metab* 2019, **16**:100095.
